# Supplementary figures and images for: Species-Specific Stress Responses to Selenium Nanoparticles in Pseudomonas aeruginosa and Proteus mirabilis
Source: Nanomaterials (Basel). 2025 Sep 12;15(18):1404. doi: 10.3390/nano15181404 (PMC12472632; doi:10.3390/nano15181404)

**Fig. S1. Pulsed Laser Ablation in Liquids**

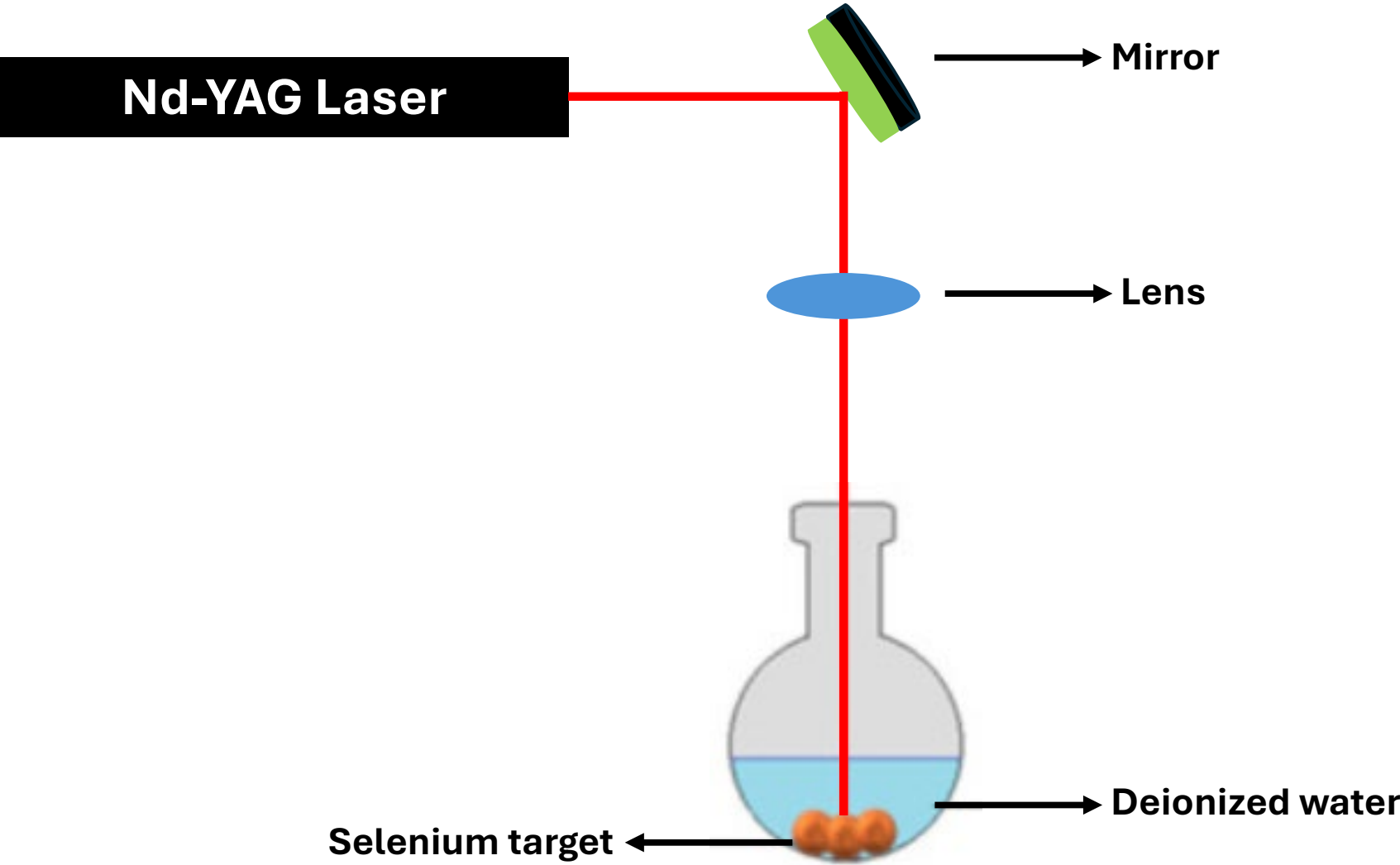

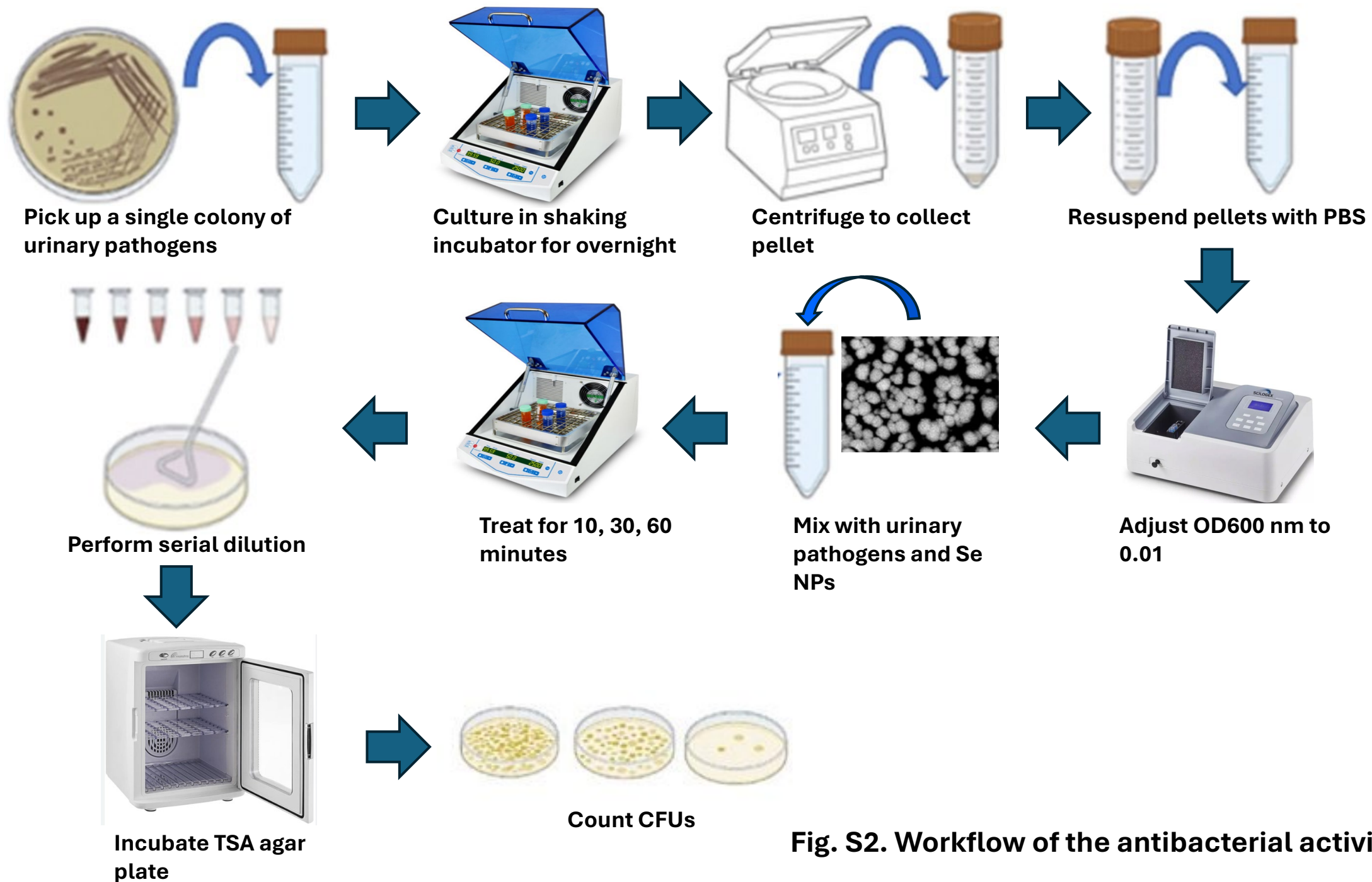

**Fig. S2. Workflow of the antibacterial activity assay**

Supplement: Supplementary file 1 [file nanomaterials-15-01404-s001.zip › nanomaterials-3832085-supplementary.pdf]
